# The Design and Optimization of Plasmonic Crystals for Surface Enhanced Raman Spectroscopy Using the Finite Difference Time Domain Method

**DOI:** 10.3390/ma11050672

**Published:** 2018-04-26

**Authors:** Alec Bigness, Jason Montgomery

**Affiliations:** Department of Chemistry, Biochemistry, and Physics, Florida Southern College, Lakeland, FL 33801, USA; abigness@flsouthern.edu

**Keywords:** finite-difference time-domain, FDTD, surface plasmons, surface enhanced Raman spectroscopy, soft nanolithography

## Abstract

We present computational studies of quasi three-dimensional nanowell (NW) and nanopost (NP) plasmonic crystals for applications in surface enhanced Raman spectroscopy (SERS). The NW and NP plasmonic crystals are metal coated arrays of cylindrical voids or posts, respectively, in a dielectric substrate characterized by a well/post diameter (*D*), relief depth (RD), periodicity (*P*), and metal thickness (MT). Each plasmonic crystal is modeled using the three-dimensional finite-difference time-domain (FDTD) method with periodic boundary conditions in the *x*- and *y*-directions applied to a computational unit cell to simulate the effect of a periodic array. Relative SERS responses are calculated from time-averaged electric field intensity enhancements at $\lambda_{\mathrm{exc}}$ and $\lambda_{\mathrm{scat}}$ or at $\lambda_{\mathrm{mid}}$ via $G_{\mathrm{SERS}}^{4}=g^{2}\left(\lambda_{\mathrm{exc}}\right)\times g^{2}\left(\lambda_{\mathrm{scat}}\right)$ or $G_{\mathrm{mid}}^{4}=g^{4}\left(\lambda_{\mathrm{mid}}\right)$, respectively, where $g^{2}={\left|E\right|}^{2}/{\left|E_{0}\right|}^{2}$. Comparisons of $G_{\mathrm{SERS}}^{4}$ and $G_{\mathrm{mid}}^{4}$ are made to previously reported experimental SERS measurements for NW and NP geometries. Optimized NW and NP configurations based on variations of *D*, *P*, RD, and MT using $G_{\mathrm{SERS}}^{4}$ are presented, with 6× and 2× predicted increases in SERS, respectively. A novel plasmonic crystal based on square NP geometries are considered with an additional 3× increase over the optimized cylindrical NP geometry. NW geometries with imbedded spherical gold nanoparticles are considered, with 10× to 10${}^{3}\times$ increases in SERS responses over the NW geometry alone. The results promote the use of FDTD as a viable in silico route to the design and optimization of SERS active devices.

## 1. Introduction

Surface enhanced Raman spectroscopy (SERS) employing metallic nanostructures is a well studied phenomenon that arises predominantly from large electric field intensity enhancements when light is incident on a metal that supports surface plasmons (SPs) [1,2,3]. SPs are collective oscillations of conducting electrons with a characteristic frequency that can exist at the interface of a dielectric and a metal [4,5]. Under certain conditions, incident light resonant with the plasmon frequency can excite the SP within the metal, giving rise to sub-wavelength field confinement and intensity enhancements, defined hereafter as $g^{2}$ = |E|${}^{2}$/$|E_{0}|$${}^{2}$, where |E| and $|E_{0}|$are the magnitudes of the total and incident electric field, respectively. (The spatial and frequency/wavelength dependence of *E* is ignored for the moment for simplicity.) The SP frequency is influenced by the size, shape, and local dielectric environment of the metal and can thereby be tuned as desired [6,7]. Molecules that traverse the SP field experience not only enhanced absorption of light proportional to the enhancement factor at the excitation frequency, $g_{\mathrm{exc}}^{2}$, but also enhanced Raman scattering proportional to the enhancement factor at the Raman scattered frequency, $g_{\mathrm{scat}}^{2}$. The overall SERS response is then proportional to $g_{\mathrm{exc}}^{2}$ × $g_{\mathrm{scat}}^{2}$ [8]. Early SERS substrates were composed of rough metal surfaces or colloidal solutions of metallic nanoparticles whose sensitivity and reproducibility suffered from the generation of only random “hot-spots” on the metal surface or in the temporal gaps between adjacent particles. Within the last decade, nanolithography methods based on soft imprinting have enabled the inexpensive fabrication of robust SERS substrates that give rise to uniform hot-spots over large areas, with enhancement factors on the order of $10^{6}$ to $10^{8}$ [9,10,11,12].

**Figure 1:**
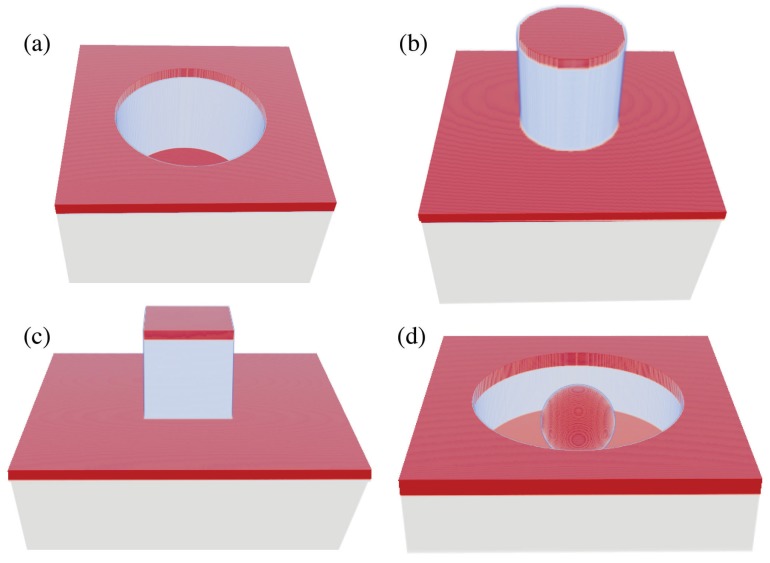
Unit cell geometries for (**a**) nanowells, (**b**) cylindrical nanoposts, (**c**) square nanoposts, and (**d**) particle imbedded nanowells. Plasmonic crystals result from the use of periodic boundary conditions.

The SERS substrates considered in this work are based on quasi-three-dimensional plasmonic crystals composed of dielectric supports patterned with either voids or posts upon which thin films of gold are deposited using electron beam evaporation. We do not consider sputtering, which can give rise to wall features. Nanowell (NW) geometries (Figure 1a) result from voids in the supporting dielectric [13], and nanopost (NP) geometries (Figure 1b) result from posts on the supporting dielectric [14]. Previous experimental studies of these NW and NP geometries involved fabricating an array of plasmonic crystals, each region of the array characterized by a particular diameter, *D*, and periodicity, *P*. The SERS response of a target molecule, namely benzene thiol, was measured for each region of the array. A finite-difference time-domain (FDTD) analysis of the geometries studied showed qualitative agreement (more on this below) between relative SERS responses, and a maximum in the SERS response was attributed to the excitation of a local surface plasmon resonance (LSPR) of the well or post.

The present work aims to re-examine these plasmonic crystal studies as a means to compare the predictive abilities of two related FDTD approaches in estimating relative SERS responses. The FDTD method is a numerical approach in which electric and magnetic field components are propagated on a set of discrete grid points, each described by a dielectric constant consistent with the material in the structure being modeled at that point [15]. FDTD is a powerful tool for modeling the interactions of light with arbitrarily shaped, complex geometric structures and has been used in a wide variety of surface plasmon applications, including sensing, photovoltaics, imaging, optical trapping, metamaterials and optical transparency, and, of course, SERS [16,17,18,19,20,21,22,23,24]. With FDTD electric field intensities calculated at both excitation and scattered wavelengths, $\lambda_{\mathrm{exc}}$ and $\lambda_{\mathrm{scat}}$, respectively, the SERS response can be calculated as

(1) $$G_{\mathrm{SERS}}^{4}=\frac{1}{N}\sum_{x_{i}}\sum_{y_{j}}\sum_{z_{k}}g^{2}\left(x_{i},y_{j},z_{k},\lambda_{\mathrm{exc}}\right)\times g^{2}\left(x_{i},y_{j},z_{k},\lambda_{\mathrm{scat}}\right),$$

where $x_{i}$, $y_{j}$, and $z_{k}$ are FDTD grid points and *N* is the number of grid points in the volume of air near the surface of a metallic nanostructure [23,25,26]. Equation (1) requires the computation of fields at $\lambda_{\mathrm{exc}}$ and $\lambda_{\mathrm{scat}}$. For large three-dimensional grids, due either to a large spatial extent or a fine grid resolution, or for long simulation times, it is possible to reduce computational cost by approximating the SERS response as a function of a single wavelength, typically $\lambda_{\mathrm{exc}}$ or $\lambda_{\mathrm{mid}}$, the midpoint between the excitation and scattered wavelenghts [13,14,27,28]. The use of $\lambda_{\mathrm{exc}}$ is suitable if the Stokes shift is small compared with $\lambda_{\mathrm{exc}}$. The use of $\lambda_{\mathrm{mid}}$ is suitable if the surface plasmon resonance is sufficiently broad over the range of excitation and scattered wavelenghts. Early studies of colloidal gold nanospheres indeed showed that particles with an LSPR parked between the excitation and scattered wavelengths were able to effectively enhance both $\lambda_{\mathrm{exc}}$ and $\lambda_{\mathrm{scat}}$ to produce the highest SERS response [29]. The SERS response based on this approximation using $\lambda_{\mathrm{mid}}$, referred to hereafter as $G_{\mathrm{mid}}^{4}$, is given by

(2) $$G_{\mathrm{mid}}^{4}=\frac{1}{N}\sum_{x_{i}}\sum_{y_{j}}\sum_{z_{k}}g^{4}\left(x_{i},y_{j},z_{k},\lambda_{\mathrm{mid}}\right).$$

The aim of this study, then, was to compare Equations (1) and (2) in estimating relative SERS responses for NW and NP plasmonic crystals, which are able to support multiple LSPRs with complicated coupling.

In Section 2.1, we show that the former approach is necessary to get more quantitative relative SERS responses. With this in mind, we then set out to design and optimize SERS substrates in silico without fabrication costs. In Refs. [13,14], the dimensions studied experimentally do not allow one to distinguish between the effect of diameter and the effect of periodicity on the SERS response, both of which can affect a SP resonance. In Section 2.2 , we optimize of NW and NP geometries with respect to well depth/post height, diameter, and periodicity using Equation (1). In Section 2.3, we present two studies looking at novel plasmonic crystals based on variations of NW and NP geometries. Differences include nanoposts with square cross-sections (Figure 1c) and nanowells with imbedded nanoparticles (Figure 1d).

## 2. Results

### 2.1. Comparison of FDTD Simulated SERS Responses for Nanowell and Nanopost Plasmonic Crystals

Time-averaged electric field intensity enhancements were calculated for λ=785, 821, and 857 nm using the finite-difference time-domain (FDTD) method for a series of nanowell (NW, Figure 1a) and cylindrical nanopost (NP, Figure 1b) geometries. For the NW geometries, an SU-8 support with refractive index n=1.59 was used with a relief depth RD=360 nm and gold metal thickness MT=40 nm [13]. For the NP geometries, an NOA support with refractive index n=1.56 was used with a relief depth RD=200 nm and gold metal thickness MT=24 nm [14]. All parameters were consistent with fabricated arrays. Periodic boundary conditions were implemented in the *x*- and *y*-directions to simulate the array. Periodicities and well/post diameters considered are listed in Table 1.

**Figure 2:**
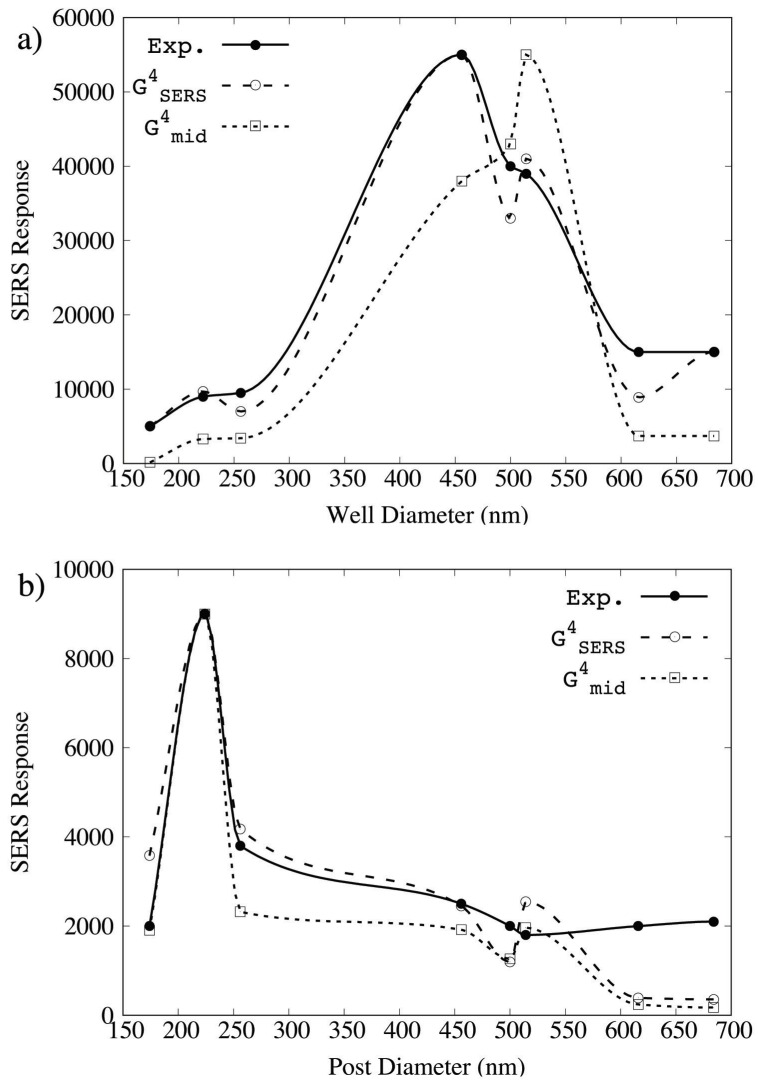
Comparison of experimental SERS response (exp) and calculated SERS responses ($G_{\mathrm{SERS}}^{4}$ and $G_{\mathrm{mid}}^{4}$) for the (**a**) NW and (**b**) NP geometries. Experimental SERS response for the NW and NP geometries are taken from Refs. [13,14], respectively. Experimental values are plotted with filled circles. SERS responses using Equation (1) are plotted with open circles. SERS responses calculated using Equation (2) are plotted with open squares. Solid and dashed/dotted lines correspond to spline interpolation between data points and are meant for ease of visualization only. Calculated SERS responses are scaled such that maximum values equal the experimental maximum.

SERS responses $G_{\mathrm{SERS}}^{4}$ and $G_{\mathrm{mid}}^{4}$ were calculated using Equations (1) and (2), respectively. Figure 2a contains a plot for the NW geometries comparing the FDTD simulated SERS responses (open symbols) with experimental measurements (filled circles) reported in Ref. [13]. (Solid and dashed/dotted lines correspond to spline interpolation between data points and are meant for ease of visualization only.)

Experimental Raman intensity measurements reveal a maximum at a diameter D=456 nm (P=730 nm) at 55,000 counts. Experimental SERS spectra were collected for a 15 mM solution of benzene thiol using a dispersive Raman microscope. The units are not directly comparable to $G_{\mathrm{SERS}}^{4}$ and $G_{\mathrm{mid}}^{4}$, which are based on unitless electric field intensity enhancements. The FDTD SERS responses, therefore, are scaled such that the maximum experimental and theoretical values coincide. It is clear from Figure 2a that $G_{\mathrm{SERS}}^{4}$, using Equation (1), reproduces the experimental SERS responses better than $G_{\mathrm{mid}}^{4}$, using Equation (2). Differences between the $G_{\mathrm{SERS}}^{4}$ and $G_{\mathrm{mid}}^{4}$ results can be understood in terms of the exictation of different LSPRs for D=456 nm and D=514 nm at 785 nm, 821 nm, and 857 nm, and predicted SERS response based on $\lambda_{\mathrm{mid}}$ alone can be misleading.

**Figure 3:**
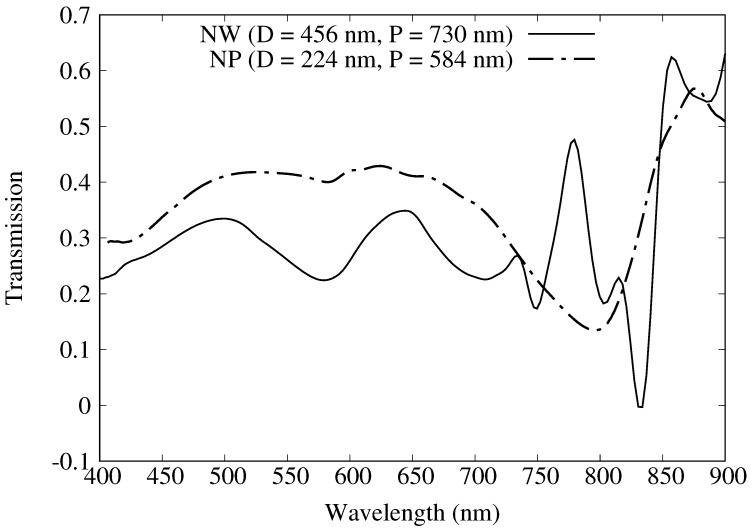
Plot of transmission for nanowell (solid) and nanopost (dashed).

In a similar comparison, Figure 2b contains a plot for the NP geometries comparing the FDTD simulated SERS responses (dashed lines with open symbols) with experimental measurements (solid line with filled circles) reported in Ref. [14]. Experimental Raman intensity measurements reveal a maximum at a diameter D=224 nm (P=584 nm) at 9000 counts, and the FDTD SERS responses are again scaled such that the maximum experimental and theoretical values coincide. (It should also be noted that the units for the experimental SERS response for the NWs and the NPs are not exactly the same, and it turns out that NP arrays produce higher SERS responses than similar NW geometries [14].) While the agreement between $G_{\mathrm{SERS}}^{4}$ and $G_{\mathrm{mid}}^{4}$ are more similar in terms of predicted SERS responses, the $G_{\mathrm{SERS}}^{4}$ values are slightly better in a least squares sense. The better agreement in the NP case can be understood as less coupling possible between the disc and the metal film, in contrast to the NW case where the void, film, and lower disc can couple, as evidenced by the differences in the transmission spectra seen in Figure 3.

### 2.2. Optimization of FDTD Simulated SERS Responses for Nanowell and Nanopost Plasmonic Crystals

**Figure 4:**
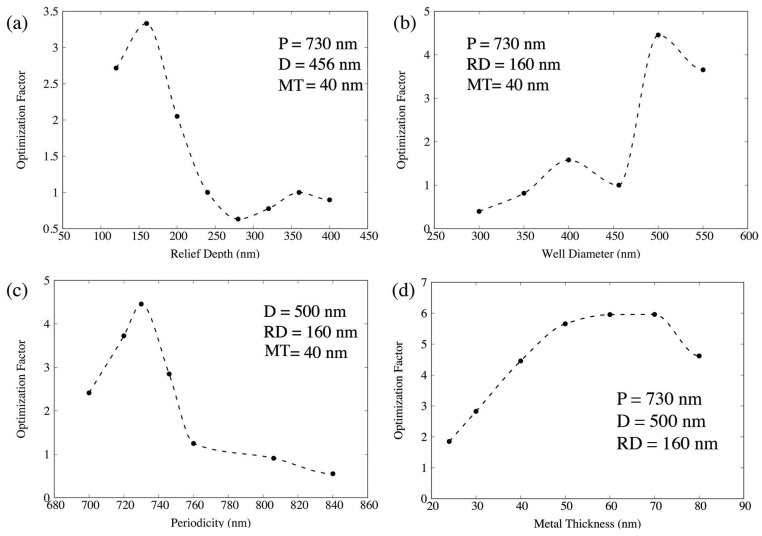
Plot of optimization factors ($O.F.\;=G_{\mathrm{SERS}}^{4}/G_{\mathrm{control}}^{4}$) as nanowell (**a**) relief depth, (**b**) diameter, (**c**) periodicity, and (**d**) metal thickness were sequentially varied. The final optimization factor is 6.0. Dashed lines correspond to spline interpolation between data points and are meant for ease of visualization only.

The better quantitative agreement between $G_{\mathrm{SERS}}^{4}$ and $G_{\exp}^{4}$ allows us to optimize relative SERS responses for the NW and NP geometries by scanning geometry parameter space, allowing the relief depth (RD), diameter (*D*), periodicity (*P*), and metal thickness (MT) to vary and calculating the resulting relative SERS response. We define the NW geometry with D=456 nm, P=730 nm, RD=360 nm, and MT=40 nm as the NW “control”. We also define an optimization factor

(3) $$O.F.\;=\frac{G_{\mathrm{SERS}}^{4}}{G_{\mathrm{control}}^{4}}$$

such that any O.F.>1 results in an enhancement of the expected SERS response. Figure 4 contains a plot of optimization factors as the nanowell RD, *D*, *P*, and MT were varied sequentially.

**Figure 5:**
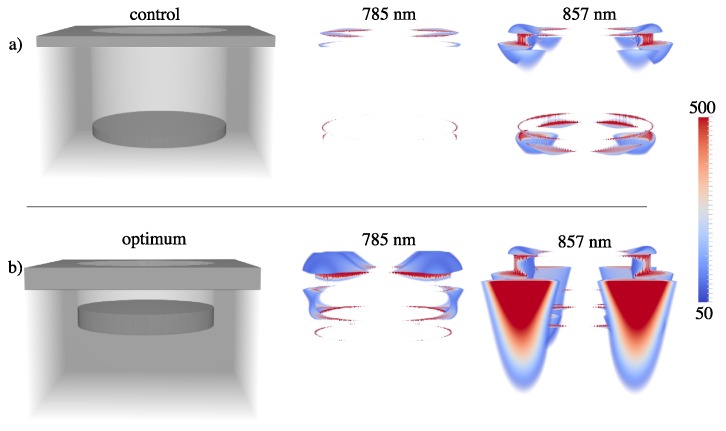
Plot of dielectric constant and time-averaged electric field intensity enhancements ($g^{2}={\left|E\right|}^{2}/{\left|E_{0}\right|}^{2}$) at λ=785 nm and λ=857 nm for (**a**) unit cell of control NW geometry (D=456 nm, P=730 nm, RD=360 nm, and MT=40 nm) and (**b**) unit cell of optimized NW geometry (D=500 nm, P=730 nm, RD=160 nm, and MT=70 nm). Only enhancements in the 50–500 range are plotted.

The final optimization factor is 6.0, indicating a nanowell plasmonic crystal with P=730 nm, D=500 nm, RD=160 nm, and MT=70 nm would give rise to a 6× increased SERS signal over the experimentally optimum structure. Figure 5 shows a comparison of electric field enhancements at λ=785 nm and λ=857 nm for the control (Figure 5a) and optimized (Figure 5b) arrays, respectively. Increased field intensities in the well region lead to a higher SERS response.

**Figure 6:**
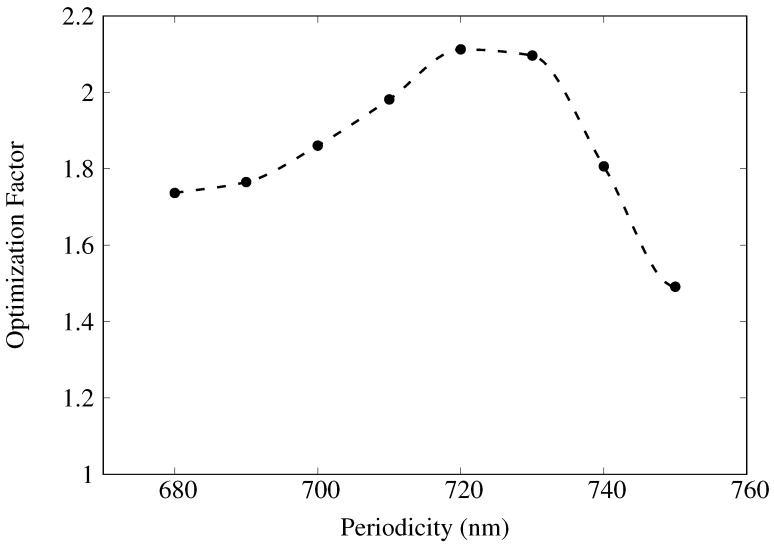
Plot of optimization factor, *O.F.*, as a function of periodicity for cylindrical NP geometry with RD=210 nm, D=200 nm, and MT=24 nm in the range 680–750 nm. Dashed lines correspond to spline interpolation between data points and are meant for ease of visualization only.

In a similar approach, the “control NP” geometry was defined as D=224 nm, P=584 nm, RD=200 nm, and MT=24 nm. By varying RD, *D*, *P*, and MT, an optimized NP geometry was determined to have D=200 nm, P=720 nm, RD=210 nm, and MT=24 nm with an O.F. =2.0. Given the similarity in the optimized and control post diameters and relief depths, the increase came predominantly through the increase in the periodicity (Figure 6).

### 2.3. Optimization of FDTD Simulated SERS Responses for Novel Plasmonic Crystals

**Figure 7:**
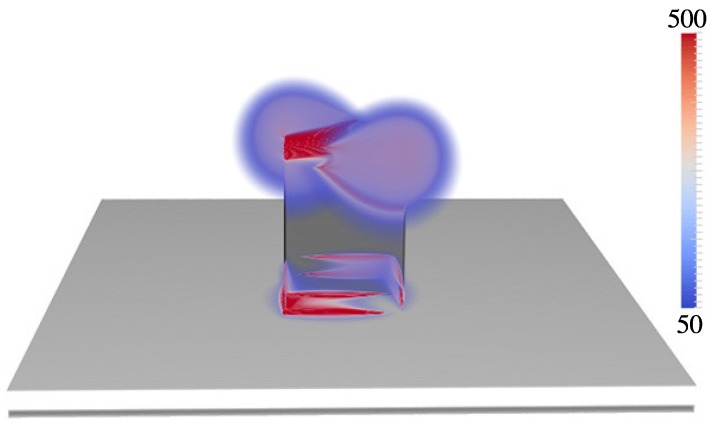
Plot of electric field intensity enhancement ($g^{2}={\left|E\right|}^{2}/{\left|E_{0}\right|}^{2}$) at λ=785 nm for optimized square NP. The optimal parameters are D=150 nm, RD=190 nm, P=730 nm, and MT=24 nm with an O.F. =6.3. Only enhancements in the 50–500 range are plotted.

In this section, we describe calculations aimed at increasing relative SERS responses even further by considering new geometries that should support either increased field intensities and/or additional coupling at 785 nm and 857 nm. To this end, we considered a square nanopost (Figure 1c) array and a nanowell array with imbedded nanoparticles (Figure 1d). The motivation for considering the square post was to excite local surface plasmons at the sharper corners, and such an array could easily be fabricated using the same soft-nanolithography techniques used to construct the cylindrical nanoposts considered above. The square NPs were positioned such that edges of length *D* were oriented along the *x*- and *y*-directions. In order to couple into surface plasmons at the corners, we used incident light polarized at 45${}^{o}$. We considered the following range of values: D=130-500 nm, RD=150-260 nm, P=500-800 nm, and MT=6-20 nm. An optimum square NP was found to have an optimization factor of O.F. =6.3 with D=150 nm, RD=190 nm, P=730 nm, and MT=24 nm. Figure 7 depicts the structure and fields at 785 nm. (Field distributions at 857 nm were similar to 785 nm and are not presented here.)

**Figure 8:**
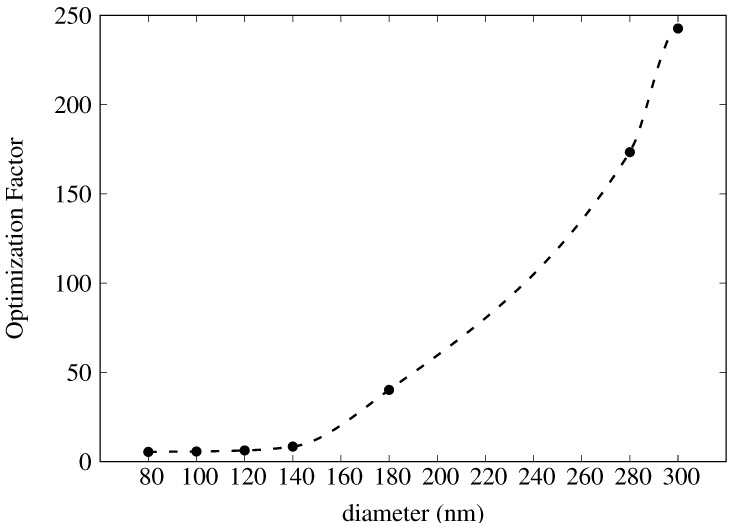
Plot of optimization factors as a function of particle diameter for spherical gold nanoparticles imbedded in an NW plasmonic crystal. Dashed lines correspond to spline interpolation between data points and are meant for ease of visualization only.

For the plasmonic crystals considered thus far, optimum SERS responses are associated with the coupling of local surface plasmons of the film and either of the void (NW) or post (NP), as indicated by periodicity and shape affect on the electric field intensities. We also considered imbedding spherical gold nanoparticles in NW geometries. The aim of this study was to increase electric field intensities by allowing coupling between the particle and the perforated film as well as between the particle and the well disc. Beginning with the optimum NW geometry from Section 2.2, we calculated $G_{\mathrm{SERS}}^{4}$ for a range of particle diameters, from 80 nm to 300 nm. Optimization factors are plotted in Figure 8. The control NW geometry is the same as used in Section 2.2, namely D=456 nm, P=730 nm, RD=360 nm, and MT=40 nm.

**Figure 9:**
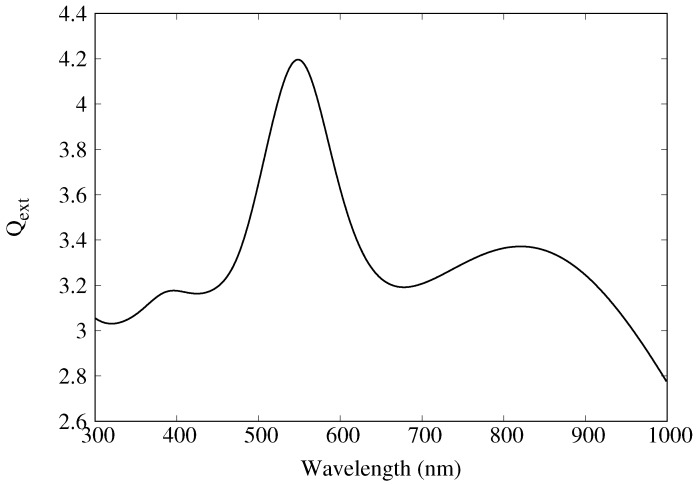
Extinction efficiency, $Q_{\mathrm{ext}}$, for a 300 nm diameter spherical gold nanoparticle.

It is not surprising that $G_{\mathrm{SERS}}^{4}$ (and hence O.F.) increases with increasing particle diameter. While isolated solid gold nanospheres do not absorb strongly in the near infrared, scattering increases for increasing particle size. Figure 9 shows the extinction efficiency (extinction cross section per geometrical cross section) for a 300 nm gold sphere. A broad peak is seen above 700 nm. The increased scattering as well as the coupling to the nanowell gives rise to increasingly large enhancements.

An initial study varying relief-depth, diameter, periodicity, and metal thickness sequentially revealed highest $G_{\mathrm{SERS}}^{4}$ values for periodicities near 730 nm and well diameters near 600 nm. We then calculated $G_{\mathrm{SERS}}^{4}$ for all combinations of *P* = 700, 730, and 760 nm, *D* = 550, 600, and 650 nm, RD = 100, 140, and 180 nm, and MT = 40 and 70 nm (see Table 2).

**Figure 10:**
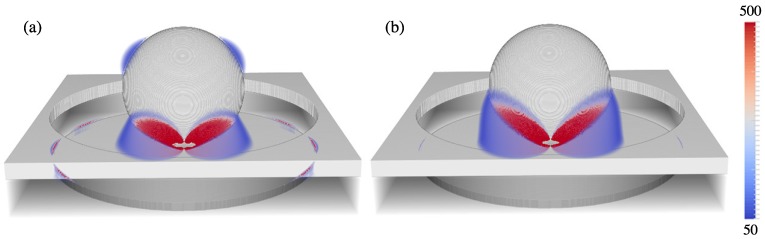
Plot of electric field intensity at (**a**) λ=785 nm and (**b**) λ=857 nm for optimized NW with imbedded 300 nm diameter nanoparticles. The optimal parameters are D=650 nm, RD=100 nm, P=700 nm, and MT=40 nm with an O.F. =2400.

Calculated O.F. values ranged from one to three orders of magnitude larger than the NW alone, with MT=40 predominantly giving rise to larger O.F. values overall. Figure 10 contains a plot of the time-averaged electric field intensity at 785 nm and 857 nm for the optimal geometry: D=650 nm, RD=100 nm, P=700 nm, and MT=40 nm with an O.F. =2400 . It is clear that the coupling of the nanoparticle to the well disc is the leading contribution to the increased $G_{\mathrm{SERS}}^{4}$. In order to assess the extent of coupling to the disc verses the film, we calculated $G_{\mathrm{SERS}}^{4}$ and associated O.F. when the metal film was removed (replaced with SU-8). The resulting O.F. =1010 shows that there is a factor of two contribution of enhancement due to the coupling of the film to the particle.

## 3. Discussion

In this work, we set out to use the FDTD method to calculate electric field intensity enhancements at $\lambda_{\mathrm{exc}}$, $\lambda_{\mathrm{mid}}$, and $\lambda_{\mathrm{scat}}$ for a series of plasmonic crystals to compare $G_{\mathrm{SERS}}^{4}$ and $G_{\mathrm{mid}}^{4}$, as defined in Equations (1) and (2), in their ability to reproduced experimental measurements. By using the word *relative*, we mean that maximum $G_{\mathrm{SERS}}^{4}$ and $G_{\mathrm{mid}}^{4}$ are scaled to match maximum experimental values. The scaling translates the unitless electric field intensity enhancement, $g^{2}$, into units consistent with experimental SERS measurements, and as structure parameters are varied, increases or decreases in the SERS response can be predicted. For both geometries, $G_{\mathrm{SERS}}^{4}$ understandably gives rise to better quantitative agreement with experimental SERS measurements. For the NP geometry, $G_{\mathrm{mid}}^{4}$ is a good approximation to $G_{\mathrm{SERS}}^{4}$ due to the broad spectral feature in the $\lambda_{\mathrm{exc}}$–$\lambda_{\mathrm{scat}}$ range (Figure 3). However, for the NW geometry which supports more complex coupling between multiple SP resonances in this range, $G_{\mathrm{mid}}^{4}$ failed to produce even qualitative agreement for some arrays. These results are not surprising; after all, $G_{\mathrm{mid}}^{4}$ is an approximation, and when underlying assumptions break down, the accuracy of an approximation also suffers. However, they do suggest caution in using $G_{\mathrm{mid}}^{4}$ for predictive applications involving complex nanostructures for SERS. It is important to note that the better quantitative agreement of $G_{\mathrm{SERS}}^{4}$ comes at a computational cost given simulations at both excitation and scattering wavelengths must be considered.

It is also important to note that alternate strategies could be used to estimate SERS responses. While Equations (1) and (2) represent an average over all grid points corresponding to air in the vicinity of the metal surface, one could envision using only maximum values of $G_{\mathrm{mid}}^{4}$ or $G_{\mathrm{SERS}}^{4}$ or averaging over only values above some threshold. While we do not present the results here, we also performed estimations of SERS responses based on these two alternate approaches and found that neither case reproduced relative experimental as well as an average over all grid points in a volume of air near each NW or NP.

We also set out to design and optimize plasmonic crystals in silico by varying structure parameters, such as relief depth (RD), well/post diameter (*D*), periodicity (*P*), metal thickness (MT), and post shape to maximize excitation and/or coupling of local surface plasmons. While only modest improvements were seen for the cylindrical nanopost, we saw a 6× increase in the NW geometry and the square NP geometry over their respective control structures. For the cylindrical NW and NP geometries and for the square NP geometry, we used a “sequential” optimization scheme, which involved varying only one parameter at a time, updating that parameter with the new optimum value before proceeding to the next parameter. This sequential approach enabled us to look at a finer grid of values for each parameter, but it is important to note that better geometries might have been excluded, as changing one parameter can affect optimum values of another previously determined. One improved approach would be to perform a more self consistent approach where we cycle through sequentially until convergence of parameters. Another approach would be to use the sequential method to identify potential optimum values for each parameter and then consider all combinations of that courser set of parameters (as was done in the case of imbedded nanoparticles in this Section 2.3 for the NW with imbedded nanoparticles.) It is likely an order of magnitude improvement over the control would be seen for the the NW and square NP geometries. In regards to fabrication, it is important to note that the novel square NP geometry presented herein should both be easily be fabricated using soft-nanolithograpy [9].

By far the most dramatic increases in predicted SERS responses arise from the addition of 300 nm gold nanoparticles to the NW geometry, with optimization factors ranging from 10× to 2000×, with an average of 600× increase in the predicted SERS response. In regards to fabrication, particles up to 300 nm can be synthesized with a uniform spherical shape and narrow size distribution using a combination of chemical reduction and annealing [30]. The challenge, though, is an even distribution of particles within the wells and not on the top metal film. We believe it is possible for the following reasons. As seen in Table 2, the optimum NW geometry with imbedded particles consisted of a 700 nm periodicity and a 650 nm well. The wells, then, correspond to almost 70% of the total surface area. By controlling the concentration of 300 nm particles, configurations with a single particle in a well should be statistically favored.

Finally, while the structures herein were optimized for the benzene thiol band at 1073 cm${}^{-1}$, it is important to note that plasmonic crystals can be designed and optimized using FDTD for any particular fingerprint Raman band for any analyte of interest. The significance of this work is in showing the potential of using FDTD to design and optimize novel plasmonic crystals in lieu of a costly trial-and-error fabrication approach.

## 4. Materials and Methods

To model the interactions of light with the nanowell/nanopost plasmonic crystals, we used Meep [31], a full three-dimensional finite-difference time-domain (FDTD) solver for Maxwell’s curl equations. In the FDTD method, electric and magnetic fields are represented on a discrete, staggered grid and are propagated in time in using a leap-frog algorithm [15]. The material composition at each grid point is specified by a dielectric constant consistent with the spatial layout of each plasmonic crystal. Dielectric supports were modeled with a constant (non-dispersive) refractive index in line with photopolymers used in fabricated arrays: SU-8 (n=1.59) [13] for NW geometries and NOA (n=1.56) [14] for NP geometries. Gold was modeled using a dispersive Drude plus two Lorentzian fit of Johnson and Christy’s dielectric data [32] over 300–1000 nm:

(4) $$\epsilon_{\mathrm{Au}}=\epsilon_{\infty }-\frac{\omega_{D}^{2}}{\omega +i\omega \gamma_{D}}-\sum_{m=1}^{2}\frac{\sigma_{m}\omega_{m}^{2}}{\omega^{2}-\omega_{m}^{2}+i\omega \gamma_{m}},$$

with $\epsilon_{\infty }=4.05665$, $\omega_{D}=8.75866$ eV, $\gamma_{D}=0.04062$ eV, $\omega_{1}=2.92893$ eV, $\gamma_{1}=0.83423$ eV, $\sigma_{1}=1.09960$, $\omega_{2}=4.05286$ eV, $\gamma_{2}=1.74845$ eV, and $\sigma_{3}=2.2195$. Periodic boundary conditions in the *x*- and *y*-directions were used to simulate square arrays. Meep’s perfectly matched layers (PML) were used as absorbing boundary conditions at the top and bottom of the computational cell to prevent spurious reflections [33]. The computational box size in the *z*-direction was 1500 nm with a grid spacing of 2 nm and 60 nm of PML in both directions. Light was propagated in the *z*-direction from air from a line source at the air/PML interface. With $\theta_{src}$ defined such that $\theta_{\mathrm{src}}=0^{o}$ corresponds to an *x*-polarized light source and $\theta_{\mathrm{src}}=90^{o}$ corresponds to a *y*-polarized light source, $\theta_{\mathrm{src}}=0^{o}$ was used for all NW and cylindrical NP geometries, and $\theta_{\mathrm{src}}=45^{o}$ was used for the square NP geometries. Electric fields for λ= 785, 821, and 857 nm were propagated for 100 fs to ensure complete coupling into surface plasmons. Electric field intensities, with and without plasmonic crystals present, were averaged over the last two periods (c/λ) to get the time-averaged electric field intensity enhancement, $g^{2}$ = |E|${}^{2}$/$|E_{0}|$${}^{2}$, at each wavelength. We calculated $G_{\mathrm{SERS}}^{4}$ and $G_{\mathrm{mid}}^{4}$ (Equations (1) and (2)) by averaging over $g^{2}$ only for points in air within 100 nm above the NW or NP. Points in the gold film or dielectric support were excluded from the average since they would not be accessible to a target molecule, and the 100 nm range from the NW or NP allowed for electric fields corresponding to surface plasmons to sufficiently decay. Zero-order transmission (T) spectra were calculated by taking the ratio of the transmitted power to the incident power integrated over a plane within the dielectric support. The extinction cross section for the 300 nm gold sphere was calculated using Mie theory [5,34]. Plots of electric field intensities were generated using ParaView [35,36].

## Figures and Tables

**Table 1:** Periodicities and diameters for experimental plasmonic crystals.

Diameter (nm)	Periodicity (nm)
174	490
224	584
256	658
456	730
500	800
514	760
616	1000
685	1100

**Table 2:** Optimization factors for nanowell geometries imbedded with 300 nm spherical gold nanoparticles.

P (nm)	D (nm)	RD (nm)	*O.F.*	*O.F.*
			(MT=40 nm)	(MT=70 nm)
700	550	100	1163	917
700	550	140	401	366
700	550	180	320	213
700	600	100	1815	1171
700	600	140	619	751
700	600	180	452	460
700	650	100	2358	1405
700	650	140	887	966
700	650	180	627	616
730	550	100	870	600
730	550	140	223	307
730	550	180	147	181
730	600	100	1538	852
730	600	140	381	411
730	600	180	184	211
730	650	100	1920	1156
730	650	140	889	897
730	650	180	647	656
760	550	100	463	561
760	550	140	52	143
760	550	180	16	47
760	600	100	837	753
760	600	140	201	248
760	600	180	164	195
760	650	100	1050	880
760	650	140	347	437
760	650	180	292	351
